# Malaria in areas under mining activity in the Amazon: A review

**DOI:** 10.1590/0037-8682-0551-2023

**Published:** 2024-06-24

**Authors:** Pablo Sebastian Tavares Amaral, Klauss Kleydmann Sabino Garcia, Martha Cecilia Suárez-Mutis, Ronan Rocha Coelho, Allan Kardec Galardo, Felipe Murta, Gilberto Gilmar Moresco, André Machado Siqueira, Rodrigo Gurgel-Gonçalves

**Affiliations:** 1 Universidade de Brasília, Faculdade de Medicina, Programa de Pós-graduação em Medicina Tropical, Brasília, DF, Brasil.; 2 Secretaria de Vigilância em Saúde e Ambiente, Ministério da Saúde, Brasília, DF, Brasil.; 3 Universidade de Brasília, Faculdade de Ciências da Saúde, Brasília, DF, Brasil.; 4 Fundação Oswaldo Cruz, Rio de Janeiro, RJ, Brasil.; 5 Laboratório de Entomologia Médica, Instituto de Pesquisas Científicas e Tecnológicas do Estado do Amapá, Macapá, AP, Brasil.; 6 Fundação de Medicina Tropical Dr. Heitor Vieira Dourado, Departamento de Ensino e Pesquisa, Manaus, AM, Brasil.; 7 Universidade de Brasília, Faculdade de Ciências da Saúde, Programa de Pós-graduação em Saúde Coletiva, Brasília, DF, Brasil.; 8 Instituto Nacional de Infectologia Evandro Chagas, Fundação Oswaldo Cruz, Rio de Janeiro, RJ, Brasil.; 9 Universidade de Brasília, Faculdade de Medicina, Laboratório de Parasitologia Médica e Biologia Vetores, Brasília, DF, Brasil.

**Keywords:** Malaria, Illegal mining, Environmental Policy, Brazil

## Abstract

Deforestation and high human mobility due to mining activities have been key to the increase in malaria cases in the Americas. Here, we review the epidemiological and control aspects of malaria in the Amazon mining areas. Epidemiological evidence shows: 1) a positive correlation between illegal mining activity and malaria incidence, mostly in the Amazon region; 2) most Brazilian miners are males aged 15-29 years who move between states and even countries; 3) miners do not fear the disease and rely on medical care, diagnosis, and medication when they become ill; 4) illegal mining has emerged as the most reported anthropogenic activity within indigenous lands and is identified as a major cause of malaria outbreaks among indigenous people in the Amazon; and 5) because mining is largely illegal, most areas are not covered by any healthcare facilities or activities, leading to little assistance in the diagnosis and treatment of malaria. Our review identified five strategies for reducing the malaria incidence in areas with mining activities: 1) reviewing legislation to control deforestation and mining expansion, particularly in indigenous lands; 2) strengthening malaria surveillance by expanding the network of community health agents to support rapid diagnosis and treatment; 3) reinforcing vector control strategies, such as the use of insecticide-treated nets; 4) integrating deforestation alerts into the national malaria control program; and 5) implementing multi-sectoral activities and providing prompt assistance to indigenous populations. With this roadmap, we can expect a decrease in malaria incidence in the Amazonian mining areas in the future.

## INTRODUCTION

**FIGURE 1:**
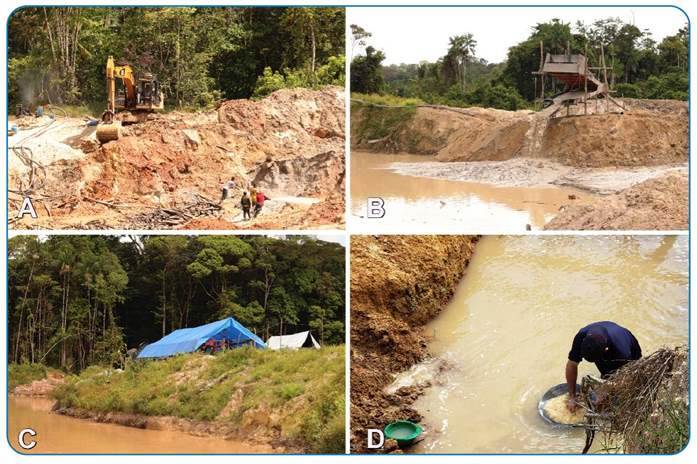
Illegal mining activities in the Brazilian Amazon region, Garimpo do Lourenço, Calçoene-Amapá. The images show mining activities in the Brazilian Amazon region, displaying an excavator **(A)**, a washing equipment (also known as Curimã) **(B)**, a housing structure **(C)**, and artisanal mining utilizing a tray (also known as bateia) **(D)**. Photos by MORESCO, G and AMARAL, PST.

Malaria is an infectious disease caused by *Plasmodium* spp. parasites that are transmitted by *Anopheles* spp. mosquito bites. By 2022, there were an estimated 249 million cases of malaria worldwide, resulting in 608,000 deaths, mostly in Africa^1^. The Amazon has the highest risk of malaria transmission in the Americas, particularly in Brazil, Venezuela, Peru, and Colombia^2^. Since 2000, the incidence (-72.5%) and mortality (-70%) due to malaria has been observed to decline in the Americas^1^. The spread of malaria is influenced by multiple factors, such as climate, deforestation, vector competence, population movement, and the organization of health services^1^ ^-^ ^5^. Deforestation and illegal mining activities are the key contributors to the increase in malaria in Americas^2^ ^,^ ^6^ ^-^ ^9^. Artisanal mining is defined as ‘individual or organized group work using basic instruments, manual tools, or portable machines’^10^. In this study, we use the term 'illegal mining' to refer to all unauthorized mining activities, including both artisanal and gold mining, because our research shows that approximately 90% of illegal mining is related to gold mining^11^. Mining activity modifies the natural landscape and results in harmful environmental impact (Figure 1), such as deforestation, river siltation, and mercury contamination of soil and water^12^ ^-^ ^14^.

Several studies have indicated a correlation between environmental changes and an increased incidence of malaria^15^ ^-^ ^18^. Furthermore, mobile populations, such as gold miners are at a higher risk of malaria infection. When miners return home, they introduce new *Plasmodium* spp. strains in vulnerable areas^2^ ^,^ ^19^ ^-^ ^27^. The impact of illegal mining activities has been reported in Brazil^22^ ^,^ ^24^ ^,^ ^28^ ^-^ ^33^, Colombia^21^ ^,^ ^34^ ^,^ ^35^, Peru^36^, Guyana^37^, and French Guiana^38^. Limited economic resources, restricted access to healthcare, and the association of mining with illicit activities, such as drug trafficking and violence, are common problems throughout the region. Illegal mining has a negative impact on the ecosystems and exposes the workers to mosquito bites, especially in the Amazon region, where access to healthcare is limited owing to its remoteness. Furthermore, the specific behaviors of gold miners (high mobility, no fear of malaria) and cross-border malaria (Venezuela-Guyana-Brazil) are the major barriers to the mining areas^20^ ^,^ ^28^ ^,^ ^39^.

## METHODS

Here, we review the epidemiological and control aspects of malaria in the Amazon mining areas. Our review included epidemiological data on malaria from special groups obtained from the Brazilian Amazon region using the Epidemiological Surveillance Information System (Sivep-Malária) and the Notifiable Diseases Information System (SINAN), the official Health Information System for malaria case investigation and notification from the Ministry of Health of Brazil. The Sivep-Malaria data are considered robust and reliable and are utilized by the government and researchers in the field. The National Malaria Prevention and Control Program (NMCP) faces challenges in accessing the mining areas, but has a strong capacity to detect cases in these areas. Although the system and case reports may be subject to errors and underreporting, the data used in this context are reliable despite these limitations^40^ ^,^ ^41^.

We analyzed the main articles published on malaria related to mining activity in the Amazon, indigenous areas, and associated control measures. Our analysis provides a critical overview of the subjects in the region and the prospects for further studies. We included all types of references related to the topic. 

## MALARIA IN MINERS

Some studies have linked the incidence of malaria to mining activity and identified multiple factors influencing its spread, including housing conditions, location of mining sites, age, and social relations^30^ ^,^ ^42^. Duarte and Fontes^24^ observed a positive correlation between illegal mining activity and malaria incidence; an average increase in malaria incidence of 0.31 (95%CI=0.22-0.40) was estimated for each 100 kg increase in gold production per year in the state of Mato Grosso. 

**FIGURE 2:**
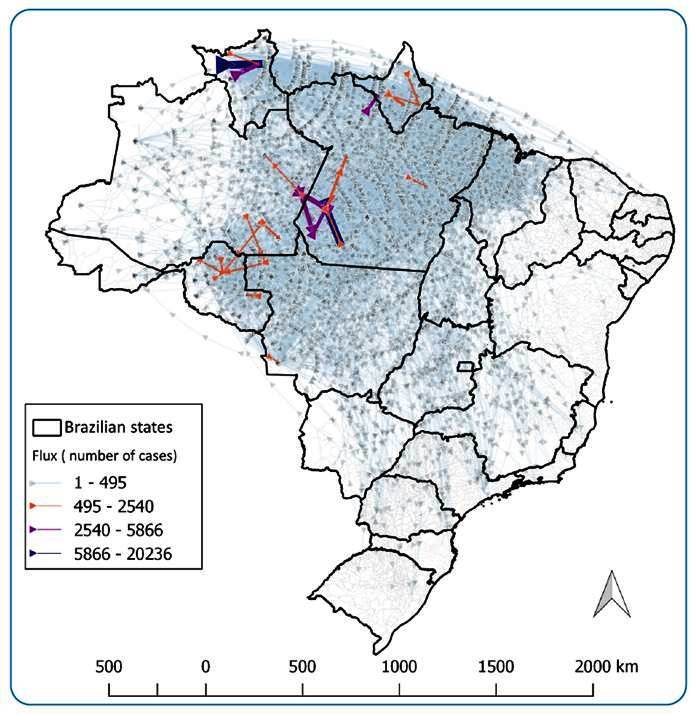
Exported cases of mining-related malaria in Brazil, 2007 to 2023. The arrow triangle is positioned at the location of the probable infection and its direction indicates the flow towards the location of detection/notification of the case. The Qgis software plugin "Flowmaps" (version 2.18) was used to analyze the flows following Garcia et al. ^44^. Source: Sivep-Malaria, Sinan - Ministry of Health.

Other studies have also shown a clear association between an increase in malaria cases and illegal mining^33^. An analysis of malaria notifications in the Brazilian Amazon region from 2011-2023 using the Sivep-Malária and SINAN systems indicated that the number of malaria cases was higher in the indigenous areas than in the mining areas. Additionally, historical data indicate a decrease in malaria cases in mining areas from 2013-2019, followed by an increase from 2020 onwards^43^. Between 2007-2022, there were 358,774 mining-related cases, accounting for 8.6% of the total cases. Of these, 268,613 cases were reported by the miners. Among all reported cases, 253,496 cases were identified in the mining areas, irrespective of occupation. In the extra-Amazon region, 992 malaria cases have been recorded among working miners. The study revealed that most individuals engaged in illegal mining were males (81.6%) aged 15-29 years (42.1%). During the analysis period, 58,163 (16.2%) of all mining-related cases were of foreign origin, with most originating in French Guiana (N=23,932; 41.1%), Venezuela (N=20,876; 35.9%), and Guyana (N=11,889; 20.4%). These three countries accounted for 97.5% of exports of mining-related cases to Brazil. Regarding the malaria cases associated with mining activities in Brazil, 113,960 cases (31.7%) were reported in municipalities other than those where the infection likely occurred. In the State of Roraima, the municipality of Alto Alegre exported the highest number of malaria cases related to mining activities to the capital, Boa Vista, totaling 20,236 cases. The highest density of flows between municipalities was mainly within the states of Roraima and Pará; however, less intense flows were also observed between Rondônia, Amazonas, and Mato Grosso. Furthermore, smaller flows spread throughout Brazil, extending from the Amazon region, mainly the state of Roraima, to the entire country, particularly to the south and southeast (Figure 2).

Mining in the Amazon is a multifaceted activity influenced by the experiences, beliefs, and cultural practices of illegal mining. Despite neglecting the risk of malaria, miners rely on medical care, diagnoses, and medications when they become ill. Access to medical treatment is frequently restricted and delayed, resulting in fatalities. The effectiveness of malaria control initiatives and the quality of available or illegal medications are in question^33^. Non-adherence to treatment is a significant concern, and financial insecurity is a major factor in treatment abandonment. The miners cease to earn money if they leave the mining areas for an extended period, forcing them to choose between seeking proper medical treatment and maintaining their income. Another factor that leads to treatment cessation is the use of illegal drugs; the belief that symptoms disappear implies that they are cured^45^. The Malakit Project (see details below) has proven to be effective in reducing malaria transmission at illegal mining sites in French Guiana. The project distributes kits to the rest areas, specific neighborhoods in border towns, and small informal settlements along border rivers that miners use to rest, buy supplies, or sell gold. Participation in interventions has been associated with significant improvements in self-care practices and a reduction in the frequency and incidence of malaria in French Guiana and Brazil^46^ ^,^ ^47^.

To customize the communication approaches and educational resources for this group, it is crucial to understand the miners’ perspectives regarding malaria. Health education is essential for miners to understand their role in malaria elimination programs. Additionally, enabling them to self-diagnose and manage their treatment in isolated mining areas using an understandable language for illiterate people is fundamental. Empowering miners improves their health and helps control malaria, thereby preventing disease outbreaks in mining regions. By comprehending the perspectives of the miners, implementing interventions, such as Malakit, and empowering these laborers, Brazil can make substantial strides in eliminating malaria in the mining communities, while concurrently enhancing the health and welfare of these individuals^45^.

## ILLEGAL MINING IN INDIGENOUS AREAS

Several indigenous populations live in forested and malaria-endemic areas of the Amazon region, resulting in an increased exposure to malaria. Moreover, illegal mining is a key factor in the spread of malaria to indigenous lands^20^ ^,^ ^27^. In 2022, the indigenous areas accounted for 30% of all locally transmitted malaria cases in Brazil^48^. Braz, Duarte, and Tauil^49^ found that indigenous people in the Amazon were twice as likely to be infected with malaria than non-indigenous people. Lapouble *et al.* ^50^ also found this in their analysis of malaria incidence. Each ethnic group has unique cultural characteristics, including dynamics that affect the mobility of its population^28^ ^,^ ^51^ ^,^ ^52^. Consequently, it has become increasingly difficult for healthcare teams to provide a precise diagnosis and adequate treatment, resulting in an increasing number of cases.

Silva-Junior *et al.* ^53^ reported a 195% increase in deforestation in indigenous territories (ITs) during the 2019-2021 triennium compared to the previous period. Several deforestation and mining activities have occurred in the ITs. In 2021, the area of illegal mining in the ITs was 102.5% higher than in 2018^27^. Illegal mining has emerged as the most frequently reported anthropogenic activity in the ITs, directly affecting *Plasmodium* spp*.* transmission^6^ ^,^ ^8^ ^,^ ^54^. The presence of infected humans and mosquito vectors in the receptive environments facilitates the spread of malaria in ITs. In addition, mining activities have created breeding sites that facilitate the reproduction of *Anopheles* spp*.* mosquitoes^45^. Wetzler *et al.* ^32^ attribute the increase in malaria cases among the indigenous communities of Munduruku, Yanomami, and Kayapó to illegal mining activities. Early childhood age groups are more susceptible to malarial infections in the ITs. Over the last three years, children between 0-9 years of age have accounted for an average of 37% of all malaria cases in ITs, according to SIVEP-Malária^55^. Moreover, more than 1,600 cases have been reported in children under one year of age in the Amazon region, based on the probable location of infection. Cases in this age group are particularly associated with recurring episodes of malaria and increased clinical severity^56^, which can have a significant impact on the health and cognitive development of affected children, as well as the well-being of the community. Indigenous communities also suffer from other health problems, such as respiratory diseases, malnutrition due to mercury contamination of water/food, neurological diseases related to mercury poisoning, and violence, especially in the Yanomami indigenous territory^33^, the largest in Brazil, where the third largest area of illegal mining in indigenous lands is located^28^. These diseases have a direct impact on malaria surveillance activities, such as the aggravation of cases and difficulty in accessing health teams.

In addition to environmental issues, malaria remains a major problem in IT because of its remoteness and fragile healthcare system. Illegal mining in indigenous areas poses a significant risk because of the potential for malaria transmission. It is important to note that mining in these areas can have severe consequences on the health and well-being of indigenous communities. Therefore, it is crucial to map these areas to enable government agencies, including those responsible for environmental, health, and indigenous issues, to take more intensive and effective action^28^ ^,^ ^31^ ^,^ ^33^ ^,^ ^43^.

## CONTROL MEASURES IN MINING AREAS

## DIAGNOSIS AND TREATMENT

As mining is largely illegal, most areas are not covered by healthcare facilities or activities, leading to fewer means of assistance for malaria diagnosis and treatment. This situation leads to extended delays in diagnosis, circulation of counterfeit drugs, use of antimalarials without confirmation of infection, and reduced adherence to treatment. The miners at mining sites frequently turn to presumptive treatment with antimalarials whenever they have fever because they lack access to a diagnosis. However, the high cost of antimalarials in the black market may cause individuals to discontinue treatment once their fever subsides and save medication for future episodes. This behavior can result in poor adherence and erratic drug exposure, increasing the risk of antimalarial resistance emergence^57^. This situation is illustrated by the high proportion of *P. falciparum* in Roraima, where a high number of mining-related cases contribute to the local epidemiology, in contrast to the rest of the country. It is known that *P. falciparum* gametocytemia is detectable only five days after symptom onset^41^ ^,^ ^58^ ^,^ ^59^, deeming it highly responsive to prompt diagnosis and treatment, with increases being observed when a delayed diagnosis occurs. The state has observed a high number of deaths owing to the combination of a high proportion of *P. falciparum* and a delay in antimalarial treatment. For instance, the average number of malaria-related deaths in Roraima differed significantly between the periods of 2013-2017 (3.2 deaths) and 2018-2022 (20), according to the Mortality Information System, Ministry of Health, Brazil.

Strategies to promote wide testing and treatment in areas with mining activity are fundamental to malaria control^28^ considering that the estimated incidence of asymptomatic infections in mining areas is approximately 20%^22^ ^,23,^ ^60^. Asymptomatic malaria is commonly observed among young adult men residing in highly endemic areas who have experienced multiple episodes of malaria^61^ ^-^ ^63^. Individuals appear to develop clinical immunity^64^ ^,^ ^65^, which prevents symptom onset, despite parasitemia. In the American continent, individuals generally exhibit low parasite burdens^61^ ^,^ ^62^ ^,^ ^66^ ^-^ ^68^.

The diagnosis of asymptomatic *Plasmodium* infections presents a challenge for healthcare systems^69^ ^,^ ^70^. Despite being commonly acknowledged as the gold standard for detecting malaria, thick blood smears have poor sensitivity for identifying individuals with a low parasite load. Less than 20% of these infections can be detected by blood smears^61^ ^,^ ^62^ ^,^ ^69^ ^,^ ^71^ ^,^ ^72^. Therefore, molecular techniques can serve as alternative tools for detecting low parasitemia^73^ ^,^ ^74^. Whole blood samples are required to use these techniques; however, the accurate collection and preservation of samples under mining conditions remains a challenge. The development of DNA extraction kits from filter paper samples has overcome the challenge of collecting biological samples from remote areas and facilitated diagnostic polymerase chain reaction (PCR) analysis in advanced laboratories^75^. The use of PCR testing in mining areas, whether legal or illicit, is not an optimal approach for epidemiological surveillance teams, given the time and instrumentation required for the procedure. Consequently, the use of rapid diagnostic tests (RDTs) and blood smear analyses is a more efficacious and suitable methodology. The World Health Organization (WHO) has been advocating the use of malaria RDTs for the clinical diagnosis of malaria. These tests have been demonstrated to have high sensitivity and specificity for symptomatic cases^76^ and are important tools for improving case management, particularly in areas with limited access^77^. One caveat is that tests may remain positive for up to one month after treatment^78^; therefore, accurate history should be taken at the time of evaluation.

The Ministry of Health recommends treating all individuals with parasitic infections with specified treatments^79^. Although there is currently no consensus on whether patients with low parasite counts, detected exclusively through molecular tests^80^ should be treated, mass drug administration is a possible intervention to combat infections^81^.

## VECTOR CONTROL

Several *Anopheles* spp. are vectors of malaria in the Americas^2^. *Nyssorhynchus* (*Ny.*, formerly *Anopheles*) *darlingi* ^82^ is a highly efficient anthropophilic species that bites indoors and transmits both *Plasmodium falciparum* and *P. vivax* making it the main malaria vector in the Brazilian Amazon region^83^ ^-^ ^85^. Deforestation and stagnant water have facilitated the spread of *Ny. darlingi* mosquitoes in the mining areas^2^. Vector control plays a vital role in the prevention, control, and elimination of malaria in transmission-prone regions^86^ ^-^ ^89^.

In 2009, the Ministry of Health in Brazil published the Guide for Local Management of Malaria Control - Vector Control^90^, which offers essential recommendations on methodologies for controlling malaria vectors in the country. The guidelines support the principles of selective and integrated control to manage vectors, community participation, and adjusted measures depending on the unique eco-epidemiological situation in each municipality.

The key aspect of selective and integrated control hinges on the timely incorporation of epidemiological data and the establishment of a consistent entomology work routine that employs comprehensive monitoring parameters to facilitate decision-making. The Brazilian Ministry of Health recommends four strategies for controlling malaria vectors in Brazil. These include management of the breeding sites through environmental control or the application of biolarvicides, intradomiciliary residual spraying, thermonebulization, and the utilization of long-lasting impregnated mosquito nets^90^. Insecticide products for vector control were provided free of charge to each state. However, few studies have determined that the effectiveness of these interventions in suppressing mosquito vector populations and reducing parasite transmission is insufficient^2^. Notably, owing to the difficulties in access, housing conditions, and violence in these areas, vector control strategies have not been fully applied, making it difficult to control the disease. However, when these measures are implemented and the affected communities cooperate, positive results are observed^91^.

## MALAKIT

The Malakit project is an international initiative for malaria control in illegal mining in French Guiana, which includes training people associated with mining to self-diagnose and self-treat the disease, report cases, and distributing bed nets^46^ ^,^ ^92^. The training of miners does not include reporting cases because this population lives in isolated conditions in the Amazon rainforest, with a lack of electricity supply and Internet connections.

The main objective of the Malakit project was to increase the appropriate use of antimalarials, which should only be administered after a positive test, for *P. falciparum*. The intervention accelerated the decline in the incidence of malaria by 42.9%^93^. Improvements in attitudes and practices have been observed as the incidence of malaria has declined^93^. The results of the Malakit project have been very promising; the intervention has been integrated into the Suriname national program, and its scale-up as a strategy for malaria control is currently being evaluated^94^. The project initially targeted miners with clinical malaria, with a focus on effective treatment against *P. falciparum*, which is the parasite associated with the most severe clinical symptoms. In 2022, the CUREMA project (Radical CURE for Malaria among highly mobile and hard-to-reach populations in the Guiana Shield) was implemented, with one arm aimed at reaching the *P. vivax* reservoir in these areas, and another arm that continued the Malakit project. The Malakit Project has not yet been integrated into the local malaria control program in Brazil.

## ENVIRONMENTAL LICENSING

**FIGURE 3:**
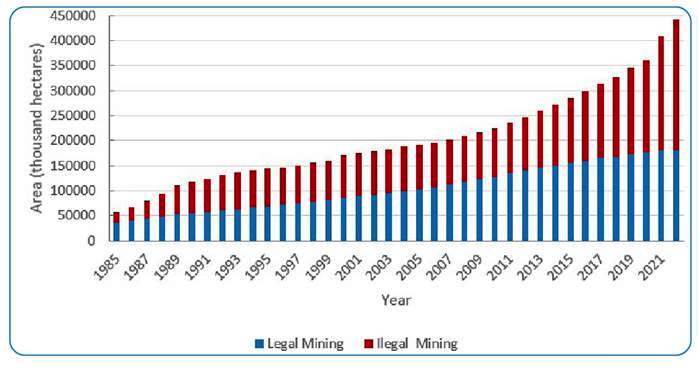
Legal mining and illegal mining areas in Brazil between 1985 and 2022. Source: MapBiomas^11^.

In 2022, 92% of the area mined in Brazil was concentrated in the Amazon region, and illegal mining was the major mining activity in the country (85.4 %). The number of mining activities in Brazil has recently increased. Within the period 1985-2022, 40.7% of the mined area has been mined in the past five years. Additionally, within the same timeframe, 62.3% of the indigenous mined land area was created. The mining area reached 442,000 ha in 2020, of which 59% were illegal mining areas. Over the period ranging from 2010 to 2022, the illegal mining areas increased by 173.96%, whereas legal mining increased by a percentage of 40.63%^11^ ^,^ ^28^ (Figure 3).

One way to combat the growth of illegal mining is to increase the monitoring and legalization of mining projects, which are already in place and robust in Brazilian legislation. However, there is a lack of political will to apply it, and these actions can no longer be delayed^28^ ^,^ ^95^ ^,^ ^96^. In the recent years, there have been no specific changes in the laws regarding environmental licensing for mining in the country. However, there has been a lenient attitude towards illegal mining, and the lack of investment and funding for policing and regulation between 2019 and 2022 has resulted in an increase in such activities. This is evident in studies that have monitored mining activities in the Amazon^17^ ^,^ ^20^ ^,^ ^28^ ^,^ ^43^ ^,^ ^97^.

The environmental licensing processes must include measures to prevent and mitigate the environmental impact of mining. In the Amazon region, targeted research is required to manage the increase in malaria cases and spread of *Ny. darlingi* ^98^ ^,^ ^99^. It is essential to assess the malaria incidence in mining regions because the decision-makers are accountable for taking appropriate measures. Understanding the impact of environmental licensing of malaria in the mining areas of the Amazon region is crucial. These findings highlight the correlation between environmental policies and public health in this area.

## PERSPECTIVES AND STRATEGIES FOR MALARIA CONTROL IN THE MINING AREAS

The number of malaria cases is declining in Brazil; however, this trend is not homogeneous across the states. The number of cases increased in Mato Grosso and Roraima between 2017 and 2021, mainly in illegal mining and indigenous localities^28^. The strategies for malaria prevention and control in mining areas should consider epidemiological data and local specificities and involve affected communities in the development process. Understanding the viewpoints of miners facilitates interventions, such as Malakit. By empowering these workers, Brazil can make significant strides in eliminating malaria in the mining communities while improving the health and well-being of these individuals. Moreover, it is important to conduct appropriate tests to detect asymptomatic individuals and screen all the individuals present in a mining area whenever a case of malaria is diagnosed. 

Controlling malaria in the mining areas can contribute to achieving Sustainable Development Goal (SDG) 3.3, which aims to end communicable diseases, such as malaria by 2030 and improve the health and well-being of the affected communities. Furthermore, successful control measures can hinder the spread of malaria to other areas, supporting the achievement of sustainable and healthy communities as directed by the SDGs. There are five key actions for controlling malaria in the mining areas in Brazil: 1) reviewing the legislation to control deforestation and mining expansion, particularly in the indigenous lands; 2) strengthening malaria surveillance by expanding the network of community health agents to support rapid diagnosis and treatment; 3) reinforcing vector control strategies, such as the use of insecticide-treated nets; 4) integrating deforestation alerts into the national malaria control program^28^; and 5) implementing multi-sectoral activities and providing prompt assistance to indigenous populations. The mining areas require specific approaches tailored to the patterns of movement, health-seeking behaviors, and environmental characteristics of the location where the exposure occurs. An integrated approach consisting of both qualitative and quantitative research is necessary to identify where control actions are required. Garcia et al.^43^ demonstrated that malaria is a significant challenge in the states of Amazonas and Roraima owing to the expansion of illegal mining and deforestation activities. Coordinated collaboration between the Environmental Health Surveillance, Health Surveillance, Public Health Emergencies Surveillance, Environmental Ministry, and Mining and Energy Ministry can play a fundamental role in strengthening malaria control in these states. We believe that, if Brazil follows this roadmap, the number of malaria cases in the mining areas of the Amazon will decrease in the coming years.

## References

[1] World Health Organization (WHO) (2023). World malaria report 2023.

[2] Recht J, Siqueira AM, Monteiro WM, Herrera SM, Herrera S, Lacerda MVG (2017). Malaria in Brazil, Colombia, Peru and Venezuela: current challenges in malaria control and elimination. Malar J.

[3] Braz RM, Guimarães RF, Carvalho OA de, Tauil PL (2014). Spatial dependence of malaria epidemics in municipalities of the Brazilian Amazon. Rev Bras Epidemiol.

[4] Molineaux L, Wernsdorfer WH, McGregor I (1988). Malaria: principles and practices of malariology.

[5] Reiner RC, Geary M, Atkinson PM, Smith DL, Gething PW (2015). Seasonality of Plasmodium falciparum transmission: a systematic review. Malar J.

[6] Chaves LSM, Conn JE, López RVM, Sallum MAM (2018). Abundance of impacted forest patches less than 5 km2 is a key driver of the incidence of malaria in Amazonian Brazil. Sci Rep.

[7] Laporta GZ, Ilacqua RC, Bergo ES, Chaves LSM, Rodovalho SR, Moresco GG (2021). Malaria transmission in landscapes with varying deforestation levels and timelines in the Amazon: a longitudinal spatiotemporal study. Sci Rep.

[8] MacDonald AJ, Mordecai EA (2019). Amazon deforestation drives malaria transmission, and malaria burden reduces forest clearing. Proc Natl Acad Sci U S A.

[9] Santos AS, Almeida AN (2018). The Impact of Deforestation on Malaria Infections in the Brazilian Amazon. Ecol Econ.

[10] Brasil (1967). Decreto-Lei n^o^ 227, de 28 de fevereiro de 1967. MINISTÉRIO DAS MINAS E ENERGIA - MME Brasil.

[11] MapBiomas (2023). Destaques do mapeamento anual de mineração no Brasil - 1985 a 2022: o avanço garimpeiro na Amazônia.

[12] Ellwanger JH, Kulmann-Leal B, Kaminski VL, Valverde-Villegas JM, DA VEIGA ABG, Spilki FR (2020). Beyond diversity loss and climate change: Impacts of Amazon deforestation on infectious diseases and public health. An Acad Bras Cienc.

[13] World Health Organization (WHO) (2007). Preventing disease through healthy environments: Exposure to mercury: A major public health concern.

[14] Vareda JP, Valente AJM, Durães L (2019). Assessment of heavy metal pollution from anthropogenic activities and remediation strategies: A review. J Environ Manage.

[15] Bauhoff S, Busch J (2020). Does deforestation increase malaria prevalence? Evidence from satellite data and health surveys. World Dev.

[16] Laporta GZ, Prado PIKL de, Kraenkel RA, Coutinho RM, Sallum MAM (2013). Biodiversity Can Help Prevent Malaria Outbreaks in Tropical Forests. PLoS Negl Trop Dis.

[17] Laporta GZ, Grillet ME, Rodovalho SR, Massad E, Sallum MAM (2022). Reaching the malaria elimination goal in Brazil: a spatial analysis and time-series study. Infect Dis Poverty.

[18] Soler LS, Verburg PH, Alves DS (2014). Evolution of land use in the Brazilian Amazon: From frontier expansion to market chain dynamics. Land.

[19] Adhin M, Labadie-Bracho M, Vreden S (2014). Gold mining areas in Suriname: reservoirs of malaria resistance?. Infect Drug Resist.

[20] Arisco NJ, Peterka C, Castro MC (2021). Cross-border malaria in Northern Brazil. Malar J.

[21] Castellanos A, Chaparro-Narváez P, Morales-Plaza CD, Alzate A, Padilla J, Arévalo M (2016). Malaria in gold-mining areas in Colombia. Mem Inst Oswaldo Cruz.

[22] de Andrade ALSS, Martelli CMT, Oliveira RM, Arias JR, Zicker F, Pang L (1995). High Prevalence of Asymptomatic Malaria in Gold Mining Areas in Brazil. Clin Infect Dis.

[23] Douine M, Musset L, Corlin F, Pelleau S, Pasquier J, Mutricy L (2016). Prevalence of Plasmodium spp. in illegal gold miners in French Guiana in 2015: a hidden but critical malaria reservoir. Malar J.

[24] Duarte EC, Fontes CJF (2002). Associação entre a produção anual de ouro em garimpos e incidência de malária em Mato Grosso - Brasil, 1985-1996. Rev Soc Bras Med Trop.

[25] Moreno JE, Rubio-Palis Y, Martínez ÁR, Acevedo P (2014). Evolución espacial y temporal de la malaria en el municipio Sifontes del estado Bolívar, Venezuela. 1980-2013. Bol Malariol Salud Ambient.

[26] Parker BS, Paredes Olortegui M, Peñataro Yori P, Escobedo K, Florin D, Rengifo Pinedo S (2013). Hyperendemic malaria transmission in areas of occupation-related travel in the Peruvian Amazon. Malar J.

[27] Wangdi K, Wetzler E, Marchesini P, Villegas L, Canavati S (2022). Cross-border malaria drivers and risk factors on the Brazil-Venezuela border between 2016 and 2018. Sci Rep.

[28] Castro MC, Peterka C (2023). Malaria is increasing in Indigenous and artisanal mining areas in the Brazilian Amazon. Nat Med.

[29] Mataveli G, Chaves M, Guerrero J, Escobar-Silva EV, Conceição K, de Oliveira G (2022). Mining Is a Growing Threat within Indigenous Lands of the Brazilian Amazon. Remote Sens.

[30] Barbieri AF, Sawyer DO (2007). Heterogeneity of malaria prevalence in alluvial gold mining areas in Northern Mato Grosso State, Brazil. Cad Saude Publica.

[31] Martins-Filho PR, Damascena NP, Araujo APD, Silva MC, Santiago BM, Deitos AR (2023). The devastating impact of illegal mining on indigenous health: a focus on malaria in the Brazilian Amazon. EXCLI J.

[32] Wetzler EA, Marchesini P, Villegas L, Canavati S (2022). Changing transmission dynamics among migrant, indigenous and mining populations in a malaria hotspot in Northern Brazil: 2016 to 2020. Malar J.

[33] de Aguiar Barros J, Granja F, Pequeno P, Marchesini P, Ferreira da Cruz M de F (2022). Gold miners augment malaria transmission in indigenous territories of Roraima state, Brazil. Malar J.

[34] Salas D, Sánchez DY, Achury G, Escobar-Díaz F (2021). Malaria en poblaciones con ocupación minera, Colombia, 2012-2018. Biomédica.

[35] Villar D, Schaeffer DJ (2019). Disarmament is the New War, Gold is the New Opium, and Ecohealth is the Historic Victim. Environ Health Insights.

[36] Sanchez JF, Carnero AM, Rivera E, Rosales LA, Baldeviano GC, Asencios JL (2017). Unstable Malaria Transmission in the Southern Peruvian Amazon and Its Association with Gold Mining, Madre de Dios, 2001-2012. Am J Trop Med Hyg.

[37] Yan SD, Orkis J, Khan Sohail S, Wilson S, Davis T, Storey JD (2020). Digging for care-seeking behaviour among gold miners in the Guyana hinterland: a qualitative doer non-doer analysis of social and behavioural motivations for malaria testing and treatment. Malar J.

[38] de Santi VP, Girod R, Mura M, Dia A, Briolant S, Djossou F (2016). Epidemiological and entomological studies of a malaria outbreak among French armed forces deployed at illegal gold mining sites reveal new aspects of the disease’s transmission in French Guiana. Malar J.

[39] Ferreira MU, Castro MC (2016). Challenges for malaria elimination in Brazil. Malar J.

[40] Moreira Braz R, Luiz Tauil P, Faria Carolina, Silva Santelli A, Jesus Fernandes Fontes C, Braz RM, Tauil PL (2016). Avaliação da completude e da oportunidade das notificações de malária na Amazônia Brasileira, 2003-2012. Epidemiol Serv Saúde.

[41] Oliveira-Ferreira J, Lacerda MV, Brasil P, Ladislau JL, Tauil PL, Daniel-Ribeiro CT (2010). Malaria in Brazil: an overview. Malar J.

[42] Ferreira IM, Yokoo EM, Souza-Santos R, Galvão ND, Atanaka-Santos M (2012). Factors associated with the incidence of malaria in settlement areas in the district of Juruena, Mato Grosso state, Brazil. Cien Saude Colet.

[43] Garcia KKS, Soremekun S, Abrahão AA, Marchesini PB, Drakeley C, Ramalho WM, Krishnasastry ST (2024). Is Brazil reaching malaria elimination? A time series analysis of malaria cases from 2011 to 2023. PLOS Glob Public Health.

[44] Garcia KKS, Abrahão AA, Oliveira AF de M, Henriques KM de D, de Pina-Costa A, Siqueira AM (2022). Malaria time series in the extra-Amazon region of Brazil: epidemiological scenario and a two-year prediction model. Malar J.

[45] Murta FLG, Marques LLG, Santos APC, Batista TSB, Mendes MO, Silva ED (2021). Perceptions about malaria among Brazilian gold miners in an Amazonian border area: perspectives for malaria elimination strategies. Malar J.

[46] Longchamps C, Galindo MS, Lambert Y, Sanna A, Mutricy L, Garancher L (2022). Impact of Malakit intervention on perceptions, knowledge, attitudes, and practices related to malaria among workers in clandestine gold mines in French Guiana: results of multicentric cross-sectional surveys over time. Malar J.

[47] Parent AA, Galindo MS, Bergeron-Longpré M, Lambert Y, Douine M (2022). Combatting malaria disease among gold miners: a qualitative research within the Malakit project. Health Promot Int.

[48] Brasil (2024). Boletim Epidemiológico 55(1) - Dia da Malária nas Américas - um panorama da malária no Brasil em 2022 e no primeiro semestre de 2023.

[49] Braz RM, Duarte EC, Tauil PL (2013). Characteristics of malaria epidemics in the municipalities of the Brazilian Amazon, 2010. Cad Saude Publica.

[50] Lapouble OMM, Santelli ACF e S, Muniz-Junqueira MI (2015). Situação epidemiológica da malária na região amazônica brasileira, 2003 a 2012. Rev Panam Salud Pública.

[51] Jitthai N (2013). Migration and malaria. Southeast Asian J Trop Med Public Health.

[52] Marques AC (1986). Migrations and the dissemination of malaria in Brazil. Mem Inst Oswaldo Cruz.

[53] Silva-Junior CHL, Silva FB, Arisi BM, Mataveli G, Pessôa ACM, Carvalho NS (2023). Brazilian Amazon indigenous territories under deforestation pressure. Sci Rep.

[54] Gonzalez Daza W, Muylaert RL, Sobral-Souza T, Lemes Landeiro V (2023). Malaria Risk Drivers in the Brazilian Amazon: Land Use-Land Cover Interactions and Biological Diversity. Int J Environ Res Public Health.

[55] Brasil. Ministério da Saúde. Secretaria de Vigilância em Saúde e Ambiente (2023). Dados para Cidadão Malária - Brasil. Dados Sivep-Malária, Sinan e E-SUS-VS.

[56] Aguiar MF de, Meireles BM, Monteiro WM, Gonçalves MJF (2022). Malaria in indigenous and non-indigenous patients aged under 15 years between 2007-2018, Amazonas state, Brazil. Rev Soc Bras Med Trop.

[57] Douine M, Lazrek Y, Blanchet D, Pelleau S, Chanlin R, Corlin F (2018). Predictors of antimalarial self-medication in illegal gold miners in French Guiana: a pathway towards artemisinin resistance. J Antimicrob Chemother.

[58] Gomes AP, Vitorino RR, Costa A de P, Mendonça EG de, Oliveira MG de A, Siqueira-Batista R (2011). Malária grave por Plasmodium falciparum. Rev Bras Ter Intensiva.

[59] Henry NB, Sermé SS, Siciliano G, Sombié S, Diarra A, Sagnon N (2019). Biology of Plasmodium falciparum gametocyte sex ratio and implications in malaria parasite transmission. Malar J.

[60] Douine M, Lambert Y, Musset L, Hiwat H, Blume LR, Marchesini P (2020). Malaria in Gold Miners in the Guianas and the Amazon: Current Knowledge and Challenges. Curr Trop Med Rep.

[61] Krieger H, Durlacher RR, Menezes MJ, Camargo EP, Silva LHP, Alves FP (2002). High prevalence of asymptomatic Plasmodium vivax and Plasmodium falciparum infections in native Amazonian populations. Am J Trop Med Hyg.

[62] Suárez-Mutis MC, Cuervo P, Leoratti FMS, Moraes-Avila SL, Ferreira AW, Fernandes O (2007). Cross sectional study reveals a high percentage of asymptomatic Plasmodium vivax infection in the Amazon Rio Negro area, Brazil. Rev Inst Med Trop Sao Paulo.

[63] da Silva-Nunes M, Moreno M, Conn JE, Gamboa D, Abeles S, Vinetz JM (2012). Amazonian malaria: Asymptomatic human reservoirs, diagnostic challenges, environmentally driven changes in mosquito vector populations, and the mandate for sustainable control strategies. Acta Trop.

[64] Antonelli LR, Junqueira C, Vinetz JM, Golenbock DT, Ferreira MU, Gazzinelli RT (2020). The immunology of Plasmodium vivax malaria. Immunol Rev.

[65] Studniberg SI, Ioannidis LJ, Utami RAS, Trianty L, Liao Y, Abeysekera W (2022). Molecular profiling reveals features of clinical immunity and immunosuppression in asymptomatic P. falciparum malaria. Mol Syst Biol.

[66] Coura JR, Suárez-Mutis M, Ladeia-Andrade S (2006). A new challenge for malaria control in Brazil: asymptomatic Plasmodium infection - a review. Mem Inst Oswaldo Cruz.

[67] Almeida ACG, Kuehn A, Castro AJM, Vitor-Silva S, Figueiredo EFG, Brasil LW (2018). High proportions of asymptomatic and submicroscopic Plasmodium vivax infections in a peri-urban area of low transmission in the Brazilian Amazon. Parasit Vectors.

[68] Almeida GG, Costa PAC, Araujo M da S, Gomes GR, Carvalho AF, Figueiredo MM (2021). Asymptomatic Plasmodium vivax malaria in the Brazilian Amazon: Submicroscopic parasitemic blood infects Nyssorhynchus darlingi. PLoS Negl Trop Dis.

[69] Gruenberg M, Moniz CA, Hofmann NE, Wampfler R, Koepfli C, Mueller I (2018). Plasmodium vivax molecular diagnostics in community surveys: pitfalls and solutions. Malar J.

[70] Coleman RE, Sattabongkot J, Promstaporm S, Maneechai N, Tippayachai B, Kengluecha A (2006). Comparison of PCR and microscopy for the detection of asymptomatic malaria in a Plasmodium falciparum/vivax endemic area in Thailand. Malar J.

[71] Suárez-Mutis MC, Coura JR (2006). Avaliação da confiabilidade da gota espessa em um estudo de campo conduzido em uma área endêmica de malária no Médio Rio Negro, Estado do Amazonas. Rev Soc Bras Med Trop.

[72] Zimmerman PA, Howes RE (2015). Malaria diagnosis for malaria elimination. Curr Opin Infect Dis.

[73] Hofmann NE, Gruenberg M, Nate E, Ura A, Rodriguez-Rodriguez D, Salib M (2018). Assessment of ultra-sensitive malaria diagnosis versus standard molecular diagnostics for malaria elimination: an in-depth molecular community cross-sectional study. Lancet Infect Dis.

[74] Imwong M, Hanchana S, Malleret B, Rénia L, Day NPJ, Dondorp A (2014). High-Throughput Ultrasensitive Molecular Techniques for Quantifying Low-Density Malaria Parasitemias. J Clin Microbiol.

[75] Hansson H, Saidi Q, Alifrangis M, Jensen ATR, Hviid L (2022). Malaria Immunology. Methods in Molecular Biology.

[76] World Health Organization (WHO) (2018). WHO technical consultation on research requirements to support policy recommendations on highly sensitive point-of-care diagnostics for *P. falciparum* malaria.

[77] Boyce RM, Muiru A, Reyes R, Ntaro M, Mulogo E, Matte M (2015). Impact of rapid diagnostic tests for the diagnosis and treatment of malaria at a peripheral health facility in Western Uganda: an interrupted time series analysis. Malar J.

[78] Dalrymple U, Arambepola R, Gething PW, Cameron E (2018). How long do rapid diagnostic tests remain positive after anti-malarial treatment?. Malar J.

[79] Brasil. Ministério da Saúde. Secretaria de Vigilância em Saúde (2021). Guia de tratamento da malária no Brasil.

[80] Tada MS, Ferreira R de GM, Katsuragawa TH, Martha RCD, Costa JDN, Albrecht L (2012). Asymptomatic infection with Plasmodium falciparum and Plasmodium vivax in the Brazilian Amazon Basin: to treat or not to treat?. Mem Inst Oswaldo Cruz.

[81] Alvar J, Alves F, Bucheton B, Burrows L, Büscher P, Carrillo E (2020). Implications of asymptomatic infection for the natural history of selected parasitic tropical diseases. Semin Immunopathol.

[82] Alvarez MVN, Alonso DP, Kadri SM, Rufalco-Moutinho P, Bernardes IAF, de Mello ACF (2022). Nyssorhynchus darlingi genome-wide studies related to microgeographic dispersion and blood-seeking behavior. Parasit Vectors.

[83] Laporta GZ, Linton YM, Wilkerson RC, Bergo ES, Nagaki SS, Sant’Ana DC (2015). Malaria vectors in South America: current and future scenarios. Parasit Vectors.

[84] Martins-Campos KM, Pinheiro WD, Vítor-Silva S, Siqueira AM, Melo GC, Rodrigues ÍC (2012). Integrated vector management targeting Anopheles darlingi populations decreases malaria incidence in an unstable transmission area, in the rural Brazilian Amazon. Malar J.

[85] Sinka ME, Rubio-Palis Y, Manguin S, Patil AP, Temperley WH, Gething PW (2010). The dominant Anopheles vectors of human malaria in the Americas: occurrence data, distribution maps and bionomic précis. Parasit Vectors.

[86] World Health Organization (WHO) (2019). Guidelines for Malaria Vector Control.

[87] Galardo AKR, Zimmerman R, Galardo CD (2013). Larval control of Anopheles (Nyssorhinchus) darlingi using granular formulation of Bacillus sphaericus in abandoned gold-miners excavation pools in the Brazilian Amazon Rainforest. Rev Soc Bras Med Trop.

[88] Dos Reis IC, Codeço CT, Degener CM, Keppeler EC, Muniz MM, De Oliveira FGS (2015). Contribution of fish farming ponds to the production of immature Anopheles spp. in a Malaria-Endemic Amazonian town. Malar J.

[89] Barbosa LMC, Scarpassa VM, Hlashwayo DF (2023). Bionomics and population dynamics of anopheline larvae from an area dominated by fish farming tanks in northern Brazilian Amazon. PLoS One.

[90] Brasil. Ministério da Saúde. Secretaria de Vigilância em Saúde (2009). Guia para Gestão Local do Controle da Malária, Controle Vetorial.

[91] Couto ÁA, Calvosa VS, Lacerda R, Castro F, Santa Rosa E, Nascimento JM (2001). Controle da transmissão da malária em área de garimpo no Estado do Amapá com participação da iniciativa privada. Cad Saude Publica.

[92] Douine M, Sanna A, Galindo M, Musset L, Pommier de Santi V, Marchesini P (2018). Malakit: an innovative pilot project to self-diagnose and self-treat malaria among illegal gold miners in the Guiana Shield. Malar J.

[93] Douine M, Lambert Y, Galindo MS, Mutricy L, Sanna A, Peterka C (2021). Self-diagnosis and self-treatment of malaria in hard-to-reach and mobile populations of the Amazon: results of Malakit, an international multicentric intervention research project. Lancet Reg Health - Americas.

[94] Douine M, Cairo H, Galindo MS, Vreden S, Lambert Y, Adenis A (2023). From an interventional study to a national scale-up: lessons learned from the Malakit strategy at the French Guiana-Suriname border. Malar J.

[95] Sonter LJ, Herrera D, Barrett DJ, Galford GL, Moran CJ, Soares-Filho BS (2017). Mining drives extensive deforestation in the Brazilian Amazon. Nat Commun.

[96] Albert JS, Carnaval AC, Flantua SGA, Lohmann LG, Ribas CC, Riff D (2023). Human impacts outpace natural processes in the Amazon. Science.

[97] Tucker Lima JM, Vittor A, Rifai S, Valle D (2017). Does deforestation promote or inhibit malaria transmission in the Amazon? A systematic literature review and critical appraisal of current evidence. Philosophical Transactions of the Royal Society B:. Biological Sciences.

[98] Brasil (2014). Portaria n^o^ 1, de 13 de janeiro de 2014.

[99] Brasil (2015). Portaria interministerial N^o^ 60, de 24 de Março de 2015.

